# Designing and validating an evaluation inventory for assessing teachers’ professional accountability

**DOI:** 10.1186/s40468-021-00128-3

**Published:** 2021-07-02

**Authors:** Maryam Rahmatollahi, Zohre Mohamadi Zenouzagh

**Affiliations:** English Translation and Teaching Department, Karaj Branch, Islamic Azad University, Karaj, Iran

**Keywords:** Educational accountability, Educational leadership, Professionalism, Professional accountability, Teacher accountability

## Abstract

Research has already established the boundless potential of teachers in assisting effective learning processes, and there is still a need to expand research to illustrate interrelation and connection between the construct of teachers’ professional accountability which moderates and directs student learning. To this end, a comprehensive review of the literature was conducted by the researchers to explore and extract relevant theoretical constructs to teacher accountability. A literature review was followed by structured interviews with 20 administrators, teachers, students, and parents to record perceived concepts related to teacher accountability. Content analysis of recorded interviews and thematic network analysis of literature resulted in a 30-item Likert scale. The researcher-made questionnaire was subject to reliability and validity issues. Thus, in the second phase, the questionnaire was piloted with 142 male and female EFL in-service teachers selected on the basis of the convenient sampling method. Factor analysis on data collected through this reduced the items to 29 and indicated that data on teacher accountability loaded on five components including accountability towards students (N: 7 items), parents (N: 5 items), school leadership (N: 5 items), society (N: 7 items), and the profession (N: 5 items). The results also indicated that the questionnaire enjoys sound psychometric properties of reliability (α: 0.88 ˂0.5). The upshots of this study could provide a better understanding of the concept and lead teachers to be more coherent and accountable.

## Introduction

As a shared consensus among education practitioners, teacher accountability intrigues many education researchers for several reasons. First, the effects teachers have on student learning are large and vary considerably across teachers, and therefore, research still is required to build up a comprehensive picture of interrelations and connections that moderate the depth and intensity of teacher accountability efficacy. Second, once implemented as teacher evaluation and accountability criteria, course credit and teachers’ paper qualifications are only weakly related to student learning and reforms should be made to address the challenges of teacher accountability, and third, teacher evaluation systems were failing to differentiate among teachers despite the large differences in teacher effectiveness on various grounds and from different perspectives (Kraft et al. 2020). Accountability, usually considered synonymous with answerability, responsibility, liability, and blameworthiness, is a concept of governance and ethics (Attarwala 2015). The term, as defined by Levitt et al. (2008), is concerned with “proper behavior, and it deals with the responsibilities of individuals and organizations for their actions towards other people and agencies” (p. vii). Educational accountability policies and studies defined accountability as individuals’ commitments at work and their commitment to follow their work when encountering repeated failures (Gottlieb 2020). Moreover, Greenblatt et al. (2020) claimed that the notion of accountability in the sociopolitical context of education should take various factors into account including healthcare, employment, and transportation to school administration, teacher education, and students.

How to assess and evaluate the depth and intensity of accountability, as not only the driving force but also a source on which possible problems are rooted, is vital whenever educational renewal is concerned. Teacher assessment has paramount importance because any renewal process deals with the examination of problems and the organization of procedures deployed to solve those problems. Besides, setting new goals and taking steps toward achieving them require individuals to constantly assess and reflect on the affairs involved (Greenblatt et al. 2020). Teacher evaluation, as a prominent school leadership function, is a peculiarity of great significance (Lillejord and Børte 2019). The turn of the century, in this respect, has witnessed teacher evaluation as a significant strategy serving processes involved in school improvement and accountability (Huber and Skedsmo 2016). Therefore, new approaches, in recent years, have emphasized accountability policies and highlighted student learning outcomes and the processes as the key in their policies (Murphy et al. 2013). As a drawback reported in various studies, all different types of accountability (i.e., accountability towards students, parents, schools, society, and the profession) were assessed and their impact was determined by the scores the students obtain on standardized tests (Greenblatt et al. 2020).

Even today, in some countries including Turkey, Bulgaria, Poland, Mexico, and Chile, Students’ test scores are regarded as of moderate to high importance in the process of teacher evaluation (Ryan et al. 2017). This is similarly true in value-added models for teacher evaluation in which teachers’ accountability is judged according to their students’ performance in academic contexts (Tarhan et al. 2019), that is, in case teachers are considered as a source of variance in the obtained results by students (McCaffrey et al. 2003). Therefore, they employed test-based accountability policies based on which teachers were rewarded or punished to the extent they could enhance their Students’ test scores. The Students’ test scores, in such systems, are used in decisions pertaining to teachers’ bonus payment and professional evaluation (Darling-Hammond 2020; Frank et al. 2020; Von der Embse et al. 2015). Judging teacher effectiveness on the basis of their Students’ test scores, however, would not be an expedient criterion simply because students’ performance on their tests is affected by many factors irrelevant to teacher efficacy and as stated by Arrafii (2021) reforming the assessment views from behaviorist to constructivist and finally to sociocultural view makes it clear that students’ scores are not the only criteria to assess their learning anymore, and there are many other factors needed to be involved in decision making. Consequently, teachers now have to be more accountable towards matters such as eliciting, interpreting evidence of learning, and then acting based on that evidence.

More importantly, Ryan et al. (2017) asserted that test-based accountability directly affects teacher attrition, stress, and burnout. Such accountability reforms, according to Kraft et al. (2020), play a significant role in increasing the possibility of an unfilled teaching position and reducing the number of teacher candidates who have been newly licensed. Lack of job satisfaction and security together with the decreases in autonomy are said to be the possible mechanisms for such effects. In line with what has been mentioned earlier, Erichsen and Reynolds (2020) also added that “accountability pressures partly undermine goals of improving performance and equity in public schools by sowing seeds of teacher dissatisfaction and contributing to teacher turnover, thus thwarting student achievement in struggling schools” (p. 1).

Besides, teacher peculiarities are different in the turn of the century. The twenty-first-century teachers, as stated by Bridges et al. (2018), are not merely obliged to be inquirers and learners who handle problems efficiently, but also are required to enhance their pedagogical content knowledge regarding the process of teaching and learning, be cognizant of their students’ developmental needs, and respect equity and diversity. Teachers, as professionals, should deploy theory to guide and modify their actions and should reflect on their classroom practice through which they can improve their own understanding of teaching acts.

Likewise, Bandele and Ajayi (2013) emphasized the role different groups of people such as students, teachers, parents, and community have on the success of each educational setting. That is, a good teacher needs to be accountable and work in collaboration with different people like students, other teachers, parents, and also society to be able to have a positive effect on the educational system.

In fact, in the current era, myriad measures about accountability and school-related quality, such as principal and teacher standards together with their performance indicators, manage school priorities and determine school and individual success (Keddie and Mills 2019). In such teaching and learning context, therefore, it is critical to set professional teaching features and characteristics that present quality learning experiences for students and ensure that every teacher endeavors to provide a good outcome. That is to say, determining what is expected from professional teachers and what qualities they are suggested to have in order to be considered accountable in their profession is a need. In accordance with AITSL (2011, as cited in Tuinamuana 2011), employing these features and standards would help teachers to identify their capabilities, professional objectives, and achievements more clearly since they let teachers observe their levels of professional practice, knowledge, and engagement and therefore contribute to the professionalization of their career. Møller (2009) explained that in many countries, accountability and its standards are among the main issues of educational reform. In these contexts, developing, benchmarking, and comparing teachers’ and principals’ professional standards seem fundamental to the new performance assessment, a matter which is missed from the field of teacher assessment.

By the same token, Attarwala (2015) stated that in any sector, professionalism is related to an individual’s level of accountability, and teaching, as a profession, requires all teachers to be accountable and responsible for their occupation. To Brady (2020), the conception of accountability requires teachers to hold themselves to account. In so doing, first, they are obliged to have certain professional dispositions. However, there has been little attention paid to the extent of teachers’ accountability, a point declared by Brady (2020) as “these conceptions fail to acknowledge the extent to which teachers are first and foremost accountable as such” (p. 1). Having an evaluation inventory based on which teachers’ accountability can be traced is a necessity. Roberts (2019) stated that there is only a general knowledge of what teachers are expected to do in their profession because there is no clear instruction to stick to in this regard. This study, thus, is intended to design and validate a teacher accountability inventory through a theory-driven literature review and data-driven thematic and content analysis of interviews with teachers, students, school leaders, and parents.

### Literature review

#### Teacher professionalism and accountability

Professionalism, as stated by Ryder (2015), is not a fixed attribute, but rather a process that varies for every teacher across different teaching contexts and at different times. However, all teachers are expected to have certain skills and abilities and keep them up-to-date so that they would have efficient lifelong development. That is, they are required to show high standards regarding their personal and professional conduct. Accountability, thus, can be taken into account by focusing on universal contributing factors This very quality of preparation for teaching is what Darling-Hammond (2020) calls a prominent aspect of accountability. Now or soon, educational systems affected by accountability pressures cause many challenges for the schools and teachers throughout the world (Frank et al. 2020).

Furthermore, making use of the standards of teaching, teacher evaluation mostly concentrates on the increased body of knowledge of effective teaching. Concentrating on a uniform or standard-based approach of teacher evaluation, external inspectors, or supervisors appraise teachers’ practice (Huber and Skedsmo 2016). Employing external standards on both school leaders and the teaching profession together with the necessity of being prepared for these evaluations have developed school self-evaluation (SSE) which is generally used synonymously with internal evaluation. The internal evaluation highlights the importance of distinguishing an accountability process stemming from the outside, that is, a government agency, and an accountability process stemming from inside, that is, a school network (Godfrey 2020).

Within the past decades, the authorities have exploited value-added models (VAMs) to assess teachers’ contributions to their students’ achievement (Tarhan et al. 2019). Many countries around the globe are implementing policies to assess and evaluate their teachers based on students’ performance on national educational assessments, which are not reliable as criticized by teaching researchers. In some countries, such as the USA, dramatic changes have been made in the past three decades regarding the way teacher effectiveness is measured. As the quality of the education gained more importance, landmark educational legislations, such as the “No Child Left Behind Act and the Every Student Succeeds Act”, established an “accountability era” (Darling-Hammond 2020; Frank et al. 2020; Holloway 2021; Ryan et al. 2017).

According to Bae (2018), then there was a necessity to have a “more meaningful next phase of school accountability, one that promotes continuous support and improvement rather than mere compliance and efforts to avoid punishment” (p. 3). Schools have to be considered as learning organizations that promise continuous evaluation and improvement, self-reflection, and supportive experimentation. To achieve these objectives and provide continuous support and effective educational renewal, having a multiple-measure accountability system is of vital importance (Bae 2018).

Professional and academic literature has presented different typologies of the concept of accountability some of which include moral, professional, administrative, political, and so forth. Such distinctions are the result of emphasizing the type of accountability relationship observed among specific actors and the type of data the actors require so that they would be able to make an informed decision regarding the conduct (Levitt et al. 2008). In this case, professional accountability functions as a prominent factor in many fields. Professional organizations foist their own standards for entry, present their own resources to ensure continuous learning, and also set their own standards of practice and monitor the implementation of such standards through direct observation of the practice (Gill 2017).

#### Models of accountability

For the concept of accountability, Starkey (2012) has presented the following three common models. The first is that of external accountability (also known as hierarchical or bureaucratic accountability), which endeavors to assess the performance of the public schooling sectors. This government-funded measure is a policy instrument at the local, regional, or national level. The accountability, in this model, is achieved through meeting goals and standards at a national, regional, and school, or even an individual teacher level. The second type pertains to a professional or internal model of accountability based on which teachers are reckoned to be accountable for their professional behavior and are evaluated with respect to some standards which are set by a professional person and government. They are also monitored by their peers. In the second model, students learning assessment is no more included. These two models are at the extreme ends of a pendulum. The third, which fits between the previous models (i.e., the professional and hyper-accountability models) and is used in the digital age, is evidence-based internal accountability that assumes teachers to be accountable and responsible for their students’ learning. In congruence with this model, evidence of students’ learning is not constrained to organized examinations. Linking evidence of demonstrated competency, skills, and knowledge and moving beyond the learning environment, digital learning records are among the main components of the teaching and learning process (Starkey 2012).

On the basis of the last accountability model, as Whitford and Jones (2000) discussed, teachers are required to go beyond responding to sanctions and rewards or failures and the success of students’ performance on standardized tests. In other words, this model of accountability does not attribute school success to Students’ test scores as it is believed that they provide insufficient information, rather it serves the more prominent goal of regarding schools as places in which students can produce work with high standards. In this regard and in line with the norms of the teaching profession and needs of the students, teachers, as competent professionals, should be accountable for their practice towards their colleagues and towards the public (Whitford and Jones 2000). The concept of accountability is the link between the needs of the students and the alignment required between theory, policy, and practice (Hayward, 2018). Without begin accountable, professionals cannot maintain. That is, if every individual is very well informed about the measures and procedures associated with accountability, then the quality of service delivery can be assured to a great extent (Maphosa et al. 2012).

With respect to the present conceptions of accountability, teachers should have cultivated professional dispositions so that they can hold themselves to account. These conceptions, however, cannot express to what extent teachers are accountable as such (Brady 2020). In educational contexts, it is of paramount importance to consider to what or to whom individuals are responsible for. Teachers, for instance, are accountable for their students, their subject matter, and for the wider society. They are required to not only pass something to the next generation but also a model and develop democratic values in their classroom (Brady 2020). The latest model of accountability, according to Whitford and Jones (2000), puts teachers in situations where they are accountable to employers, parents, and the public. Teacher accountability emphasizes the norms, ethics, and values of the profession. Promoting teacher responsibility for students’ outcomes is not the only concern here and other factors such as increasing teachers’ capacity to clarify these outcomes with respect to the ethics of the professional responsibility and practices that research determines for this profession are among the core considerations as well. In short, the concept of accountability addresses issues related to teaching, learning, and school improvement (Donaldson 2015).

### Factors related to teachers’ professional accountability

#### Accountability to students

“Teachers are primarily accountable to the learners” (Maphosa et al. 2012, p. 546). It is clarified as “Measurement of Student Growth” (Holloway 2021). Enabling students to progress efficiently, assisting them to achieve optimal learning goals, and generally preparing them for their future cannot be achieved without having hardworking and efficient teachers whom they can count on and trust in. The strong relationship between teacher quality and the amount of student learning makes the necessity of having effective systems of teacher accountability more clear (Kleinhenz and Ingvarson 2004). Attarwala (2015) regarded teachers and students as two main pillars of the process of teaching and learning. Learners cannot progress or develop unless they have a sympathetic, hardworking, and sincere teacher who paves the way for their learning. By the same token, Maphosa et al. (2012) asserted that as the main curriculum transactions occur between teachers and learners, teachers are obliged to be responsible for various needs of the students. As classrooms are replete with students of different needs and wants, it is on teachers’ shoulders to accommodate all students in their teaching procedures and to guarantee that every student receives quality instruction. Attarwala (2015) explained that providing students with quality programs and employing accountability strategies that are effective for obtaining meaningful results are among factors for which teachers are accountable for. Kanika (2016) added that students’ progress should be taken into account based on their capacity and therefore teachers should commit themselves to the students’ benefits as their success is dependent on teachers’ competence and their accountability and dedication.

#### Accountability to parents

In addition to students, teachers should also be accountable to the students’ parents or to the ones who are their guardians as definitely they have had faith in teachers and the school system. Although parents have different levels of literacy and different educational backgrounds, they have certain expectations from school, and in this respect, teachers should be responsive to them for their actions as they are among the key stakeholders in education (Maphosa et al. 2012). Parents, as stated by Kanika (2016), are among the most significant stakeholders seeking teachers’ indulgence in achieving their optimal goals and objectives. As a consequence, teachers and instructors must dedicate “more time in public relation, parent counseling, and behavioral therapy to fulfill the global societal needs” (p. 53(.

#### Accountability to school

Schools should be accountable for the students they serve, for the parents who trust in them, and for the state and communities where they are placed. Such a notion of accountability is robust. Weak accountability, on the other hand, means that nobody knows what schools do, why, and how. As a result, school accountability reveals that students are kept safe and learning, and the well-qualified teachers, in the school environment, play a significant role to help schools achieve these objectives (Roberts 2019). Teachers with their instructional strategies and approaches are among the main factors influencing the effectiveness and enhancement of schools (Huber and Skedsmo 2016).

#### Accountability to society

Accountability is “the serious act of a mature and stable state” (Roberts 2019, p. 87). This, however, is not achievable unless every member of the society is taken to be accountable to one another. In the same vein, teachers are obliged to go beyond their classroom environment and their school context and be accountable towards the society they live in. In this regard, Kanika (2016) maintained that teachers should act as a bridge between school and society. They must be accountable to the society to which they belong, should coordinate various activities of the society, and should inspire the deprived sections to educate. Attarwala (2015) asserted that teachers should “bring the students into the educational fold, coordinate various activities of the society and motivate the weaker sections of the society to learn because he can develop the confidence to link between the school and the society” (p. 48). Roberts (2019) also believed that in order to gain public trust in the profession and keep high standards of ethics, teachers have to show they follow high standards of both personal as well as professional conduct both inside and outside schools.

#### Accountability to profession

As teachers try to improve the whole educational system, they should bear in mind that they lie at the heart of this system, and therefore, their improvement would directly lead to the enhancement of all involved individuals. They should possess the skills and abilities required to better conduct their profession and diagnose what is right or wrong. In so doing, they should dedicate themselves to perennial teaching and learning. As Attarwala (2015) stated, “a teacher should devote his whole life to teaching as well as learning for the future of humanity as his role is multidimensional and multifarious” (p. 48). They are not only responsible for their own learning but also are heartened to assist their colleagues. As Schleicher (2018) put it, “teachers are accountable not to administrative authorities but primarily to their fellow teachers” (p. 116). Attarwala (2015) added that teachers should consider different ways and means to assist learners to obtain knowledge, enhance academic potential, and have a better teaching and learning process. Teaching and learning is a lifelong process and teachers as professionals have to keep themselves up-to-date as it is only a self-learning person who can maintain professional development (Kanika 2016).

#### The present study

There were several motives for current study. Inappropriateness of test-based, credit and certification-based, student measures of teacher accountability motivated present research. Besides, there is paucity of research on multidimensional evaluation of teacher accountability from an etic and emic perspective which increase the ecological validity of measuring instrument. In addition, with the recent change in education and more involvement of technology in education and its infancy in most Asian countries, there is a need to recognize educational accountability, its peculiarities, and ways to improve it. This study intends to design and validate an inventory to explore teacher professional accountability from dimensions of accountability to students, to parents, to schools, to the society, and finally to the profession.

## Method

To achieve the aforementioned objectives of this sequential exploratory mixed methods research in revisiting the constructs of teachers’ professional accountability and presenting a balanced measurement, a step-by-step procedure was conducted including different sections among which participant selection, instrument used, design, and data coding and analysis are noteworthy. More information on these sections is presented below.

### Participants

In conducting this research, two independent groups of individuals were involved for data collection. At the initial stage, a total of 20 male and female administrators, teachers, students, and parents ranging in age from 15 to 43 were recruited through non-probability purposeful sampling based on which representative sample elements are precisely chosen from the population (Ary et al. 2014). The participants’ individual differences were not the focus in the course of the current investigation, and they came from ethnically, socially, and culturally different sections of society so that a comprehensive picture of social responsibilities would be taken into account.

From among administrators, one was a Ph.D. holder, the other was a Ph.D. candidate, and the last one had a BA and their experience of being in an administrative position varied from 1 to 10 years. Except for one teacher, others were all Ph.D. candidates majoring in teaching English as a foreign language at the Islamic Azad University, Karaj Branch. Among the students, three were studying at school and others were university students studying for a BA degree. Moreover, all were native speakers of Farsi and were learning or teaching English as a foreign language.

Following Dornyei (2007) in stating that a sample size of 100 participants would provide sufficient information for running a factor analysis, to scrutinize the underlying constructs, as for the next stage, the researchers recruited 142 in-service teachers of both genders (129 females and 13 males) with the average age range and average teaching experience of 36 and 10 years, retrospectively. The participants’ L1 was Persian. They had different educational background and were teaching at different levels and for different local schools and institutes at Karaj district in Iran. Moreover, the criteria for their selection were their contribution to the field of teaching English as a foreign language, rather than their individual differences as probing this factor would go beyond the scope of this study.

### Materials and instruments

To understand and determine the theoretical framework involved, a comprehensive review of the related literature on teachers’ professional accountability was conducted at the onset of the study. This formed the primary steps required in developing the instrument as through the implementation of the inductive approach from the literature, the following questions were proposed:

1- How can teachers be accountable to students? What are the characteristics to be considered in this respect?

2- How can teachers be accountable to parents? What are the characteristics to be considered in this respect?

3- How can teachers be accountable to schools? What are the characteristics to be considered in this respect?

4- How can teachers be accountable to society? What are the characteristics to be considered in this respect?

5- How can teachers be accountable to the profession? What are the characteristics to be considered in this respect?

As observed, the questions take five aspects of teachers’ accountability to students, parents, schools, society, and the profession into account. Next, in an attempt to compile the items of the inventory for teachers’ professional accountability and operationalizing the indicators, structured interviews with 20 administrators, teachers, students, and parents were conducted and were simultaneously recorded. Using Nvivo software, the content of the recordings were coded and analyzed to develop a Liker scale consisting of 30 items with four alternatives including (1) never, (2) sometimes, (3) often, and (4) always. This was done following Nemoto and Beglar (2014)’s argument declaring that while constructing an instrument, one should avoid middle or neutral categories as they are disordered and will cause statistical problems. Four-point scales are easily understandable for individuals, as they need less effort to answer. In addition, to ensure the content validity, the first draft of the items was e-mailed to two experts, who were TEFL university lecturers, to be checked for its accuracy and relevance. They were asked to add comments to the items and make modification if required. The items were then piloted with 142 in-service teachers, and the results of their responses were used for the estimation of reliability reported in the result section.

### Design

**Fig. 1:**
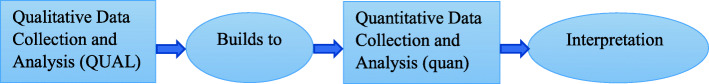
Exploratory sequential mixed methods (Creswell 2014, p. 220)

This study, as previously mentioned, was conducted through an exploratory mixed methods design. In general, the main objective of using mixed methods research is to combine qualitative and quantitative approaches in different ways and make use of their strength within one single study (Ary et al. 2014). An exploratory sequential mixed methods research is a two-phase project in which the researcher first explores the qualitative data, analyzes the results, and moves to the next step, that is, the quantitative phase based on the results of the first phase. In other words, in the case of developing a questionnaire, the researcher utilizes a three-phase procedure involving exploratory of qualitative data, development of the instrument, and then administrating the instrument to a sufficient number of participants selected as the sample of the population (Creswell 2014). The design is illustrated in Fig. 1 below.

### Procedure

**Fig. 2:**
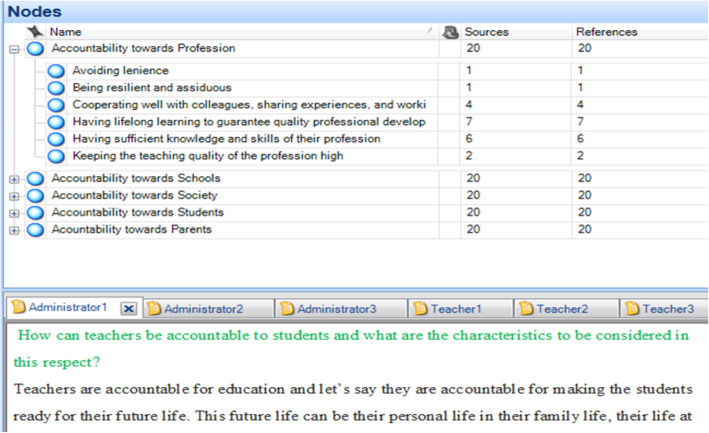
Sample screen print of the nodes specified using Nvivo software

After a thorough academic literature on teachers’ professional accountability, a theoretical framework focusing on teachers’ professional accountability towards students, parents, schools, society, and the profession was obtained through a thematic network analysis in which utmost attention was paid to factors that accountable teachers are required to have or to follow. In operationalizing the indicators and compiling the items, a total of 20 administrators, teachers, parents, and students were recruited through non-probability purposeful sampling and subsequently structured interviews were conducted by the researchers to record the perceived concepts regarding teacher accountability. The contents of the candidates’ recordings were transcribed and analyzed using Nvivo software (see Fig. 2 below).

All taken together, the researchers developed a 30-item four-point Likers scale (see Appendix A) including six items for each of the five objective groups of students, parents, schools, society, and profession. To avoid any discrepancy between respondents’ interpretation and scale intention, easy-to-understand and straight forward language was used so that no complexity, misunderstanding, and inconvenience would occur. Moreover, two experts were invited through e-mail to judge the accuracy, consistency of style, relevance, domain validity, and readability of the questionnaire and were asked to determine whether the questionnaire presented an appropriate description of the research objectives. Following their modification, in the piloting phase, 142 teachers consented to fill-out the questionnaire. Later, their responses were submitted to SPSS version 26 to quantitatively analyze the data and check the reliability of the questionnaire.

## Results

To achieve the objective of the research in designing and validating an evaluation inventory for assessing teachers’ professional accountability within the five domains of students, parents, school, society, and profession, the study comprised two stages. In the first stage, based on the reviewed research and structured interview with 20 individuals, content domains for each component were generated which ultimately led to the development of a 30-item questionnaire consisting five components with six items for each.

Then, with the cooperation of 142 teachers in the next stage, the data were collected to run a factor analysis and come up with the number of the items loaded on each component. Prior to the analysis, however, it was necessary to estimate the reliability index of the instrument through Cronbach’s Alpha. As presented in Table 1 below, the value obtained was .88 which is illustrates the strong reliability index of the questionnaire as to Hinton et al. (2014) values higher than .8 bear strong reliability.

**Table 1:** Cronbach’s alpha of the teachers professional accountability questionnaire

Reliability index		
Components of the questionnaire	Cronbach’s alpha	*N* of items
Teachers’ professional accountability self-report scale	.88	30

Analyzing the psychometric properties and ensuring the acceptable reliability value of the questionnaire, a factor analysis was run on the five components, and the relevant upshots are all reported in Tables 2, 3, and 4, as well as Fig. 1. As establishing the plausibility of a data set to be used is considered as a prerequisite of going through the main analysis, the Kaiser-Meyer-Olkin (KMO) test of sample adequacy and the Bartlett’s sphericity test were employed. In this case, Hinton et al. (2014) suggested that the KMO value greater that .5 would be optimum and would indicate that the sample collected is adequate and for the Bartlett test, the *p* value below .05 would prove that the data frame is not an identity matrix. Table 2 displays the results obtained in this regard.

**Table 2:** KMO and Bartlett’s test

Kaiser-Meyer-Olkin measure of sampling adequacy		.82
Bartlett’s test of sphericity	Approx. chi-square	1623.63
	df	435
	Sig.	.00*

**Table 3:** Total variance explained

Component	Initial eigenvalues			Extraction sums of squared loadings			Rotation sums of squared loadings		
	Total	% of variance	Cumulative %	Total	% of variance	Cumulative %	Total	% of variance	Cumulative %
1	7.49	24.97	24.97	7.49	24.97	24.97	3.47	11.57	11.57
2	3.04	10.13	35.10	3.04	10.13	35.10	3.44	11.48	23.05
3	1.80	6.00	41.11	1.80	6.00	41.11	3.04	10.16	33.21
4	1.63	5.46	46.57	1.63	5.46	46.57	2.86	9.53	42.75
5	1.39	4.66	51.23	1.39	4.66	51.23	2.54	8.48	51.23

**Table 4:** Results from a factor analysis of the items in the Teacher Accountability Questionnaire

Teacher Accountability Items	Component				
	1	2	3	4	5
**Factor 1: Accountability to Students**					
Q15. I enable schools to provide high-quality education and achieve their objectives	**.50**	.03	.46	.22	.10
Q24. I strive to provide deprived students with updated technology, libraries, laboratories, and other school resources.	.**54**	.05	.03	.27	.14
Q26. I work collaboratively with colleagues and help them improve.	**.52**	.07	.25	.28	-.04
Q27. I attempted to have sufficient knowledge and skills and take courses if necessary.	**.55**	-.05	.29	.08	.38
Q28. I promote the teaching profession by doing research.	**.78**	.04	.21	-.09	.16
Q29. I attend seminars, workshops, and other enrichment programs to be up-to-date.	**.76**	.09	.02	.04	.22
Q30. I dedicate myself to keep the quality of the profession high.	**.53**	.14	.18	.06	.51
**Factor 2: Accountability to Parents**					
Q8. I keep in touch with parents and inform them about students' academic performance.	.16	**.78**	-.09	-.03	.06
Q9. I carefully listen to parents and help them with students' affairs.	-.06	**.83**	.03	.03	.02
Q10. I make a relationship with parents based on trust and understanding.	.05	**.79**	.08	.02	.02
Q11. I work well with parents to enhance students' progress.	.18	**.77**	.18	.22	-.05
Q12. I give parents a report on the way they can accomplish their duties.	.06	**.69**	.01	.19	.08
**Factor 3: Accountability to Schools**					
Q7. I have a sense of respect for parents.	-.03	.13	.**45**	-.02	.23
Q13. I cooperate well with the administrative system.	.08	-.05	**.53**	.13	.21
Q14. I am punctual and well-disciplined.	.08	.17	**.46**	-.20	.40
Q16. I adhere to the principles and rules of schools.	.16	-.05	**.75**	.03	.12
Q17. I demonstrate decent personal behavior and attitude towards school principals and staff.	.16	-.05	**.72**	.25	-.09
**Factor 4: Accountability to Society**					
Q6. I assess students' performance objectively.	-.01	-.02	.14	**.58**	.27
Q18. I provide school principals with a report on students' performance and inform them bout students' wants and needs.	.26	.20	.47	**.47**	-.04
Q19. I teach students to act according to moral standards and cultural issues of society.	.10	.13	.36	**.50**	.06
Q20. I motivate low socioeconomic sections of society to educate.	.08	.08	.12	**.68**	.17
Q21. I endeavor to eliminate violence, discrimination, and social ills and bring equality.	.07	.11	.27	**.39**	.24
Q22. I coordinate and participate in different social affairs.	.45	.27	-.13	**.50**	-.01
Q23. I dedicate myself to students' social and emotional development	.40	.21	-.15	**.53**	.06
**Factor 5: Accountability to Profession**					
Q1. I assist students in attaining educational goals by providing maximum learning opportunities.	.11	-.07	.05	.18	**.74**
Q2. I provide students with the information they require and the materials they need.	.13	-.12	.03	.28	**.35**
Q4. I use various methods, techniques, and resources to improve students' performance.	.20	.15	.31	.10	**.46**
Q5. I encourage a sense of hard work in students and motivate them to strengthen their week points.	.12	.04	.04	.46	**.56**
Q25. I try to have lifelong learning to guarantee professional development.	.31	.20	.26	.20	**.56**

Extraction Method: Principal Component Analysis

Rotation Method: Varimax with Kaiser Normalization

^a^. Rotation converged in 10 iterations

The value of the KMO test of sample adequacy, as Table 2 illustrates, is .82 which is greater than .5 introduced as the critical value, and this implies that the collected sample was adequate (Hinton et al. 2014). Furthermore, the implication of the Bartlett’s sphericity test indicated that the *p* value was below .05, and therefore, the data frame under investigation was not considered as an identity matrix. On the basis of the fact that the KMO value is more than .6 and that the Bartlett’s test of sphericity is reckoned to be significant, one may assume the factorability of the correlation matrix.

Extraction method: principal component analysis

The total variance explained table is primarily implicated to reveal how variance is divided among factors. In this respect, a factor can be explained as having much variability in the data as a single original variable in case its eigenvalue is 1. On the contrary, if the eigenvalue is less than these common criteria, then it is declared that the factor yields information less than that of a single item (Hinton et al. 2014). The results, as observed in Table 3, show that all the five components of teachers’ accountability to students, parents, schools, society, and profession are found to be useful since their eigenvalues are 7.49, 3.04, 1.80, 1.63, and 1.39, respectively.

In the following, the scree plot of the principal components is provided to visualize the point at which the eigenvalues fluctuation almost stops. As Hinton et al. (2014) stated, factors up to that point are the significant while others are not.

**Fig. 3:**
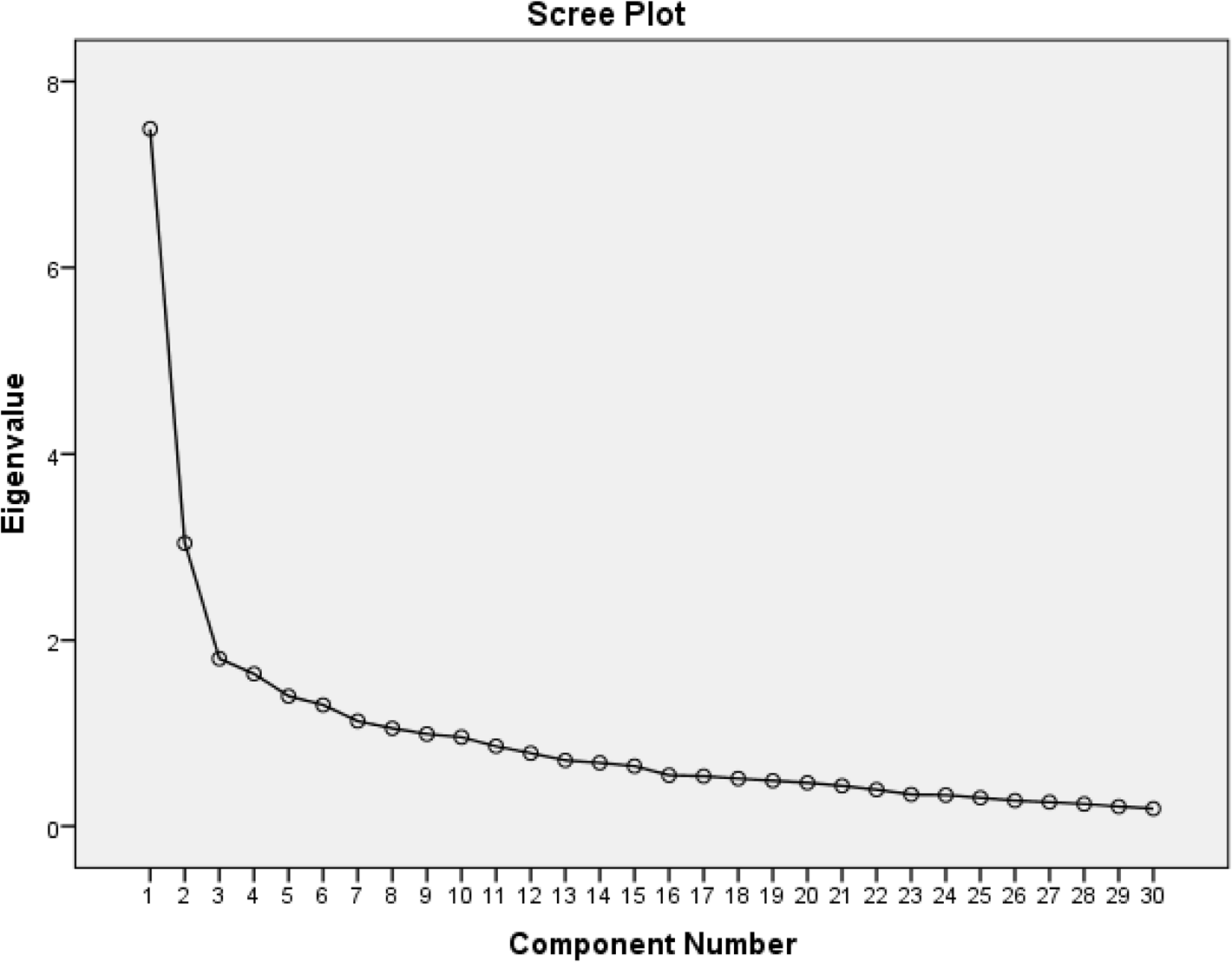
Scree plot of the eigenvalues and the items of the questionnaire

The scree plot presented in Fig. 3 suggests that the five components have significant eigenvalues, and this confirms the results found in Table 3.

Next, Table 4 is implicated to indicate item loadings on different factors. As Hinton et al. (2014) explained, variable involvement is considered significant with a case if the loading is .3 or higher. Correspondingly, as the reported values in Table 4 illustrate, seven items loaded above .3 on the first component, five items on the second, five on the third, seven on the fourth, and five on the fifth component. Besides, item 3 did not reach the desired .3 value and therefore was omitted from the questionnaire.

The results of the factor analysis aiming at probing the underlying constructs to teachers’ professional accountability, as illustrated in Table 4, indicate that the ultimate self-report scale included the following five factors along with their related items:

Factor one, named accountability to students, accounted for 24.97% of the total variance, and included seven items (i.e., 15, 24, 26, 27, 28, 29, and 30).

Factor two, named accountability to parents, accounted for 10.13% of the total variance, and involved the five items of 8, 9, 10, 11, and 12.

Factor three, called accountability to schools, accounted for 6.00% of the total variance, and included only five items (i.e., 7, 13, 14, 16, and 17).

Factor four, named accountability to society, accounted for 5.46% of the total variance, and included the following seven items: 6, 18, 19, 20, 21, 22, and 23.

Eventually, factor five, named accountability to the profession, accounted for 4.66% of the total variance, and involved the five items of 1, 2, 4, 5, and 25.

Moreover, as item selection is concerned, a problematic item, that is item 3, was removed for analysis due to its lower than .3 loading value.

## Discussion

The present study aimed at designing and validating a measurement instrument for teachers’ professional accountability. Having reviewed the related literature, the theoretical constructs regarding teachers’ accountability to students, parents, school, society, and profession were educed and were later utilized as the foundation of structured interviews that led to the development of the questionnaire. Administering the questionnaire to 142 in-service teachers and analyzing the results, it was demonstrated that the Cronbach’s alpha reliability index was estimated to be .88 indicating good internal consistency of the instrument. Additionally, the results of the factor analysis revealed no construct-irrelevant factor and all items, expect for item 3, had considerable contributions to their respective constructs. This research is assumed to formulate a deeper understanding of how to encounter challenges raised by VAMs as well as those of student surveys, classroom observation, principal evaluation, and so forth as Goe et al. (2008) put it teacher self-report measures permit the collection of a vast amount of information at once and help going through the unobservable intentions and beliefs.

Align with the objectives of having a high-quality educational setting, the development of various instrument for assessing accountability has seized scholars’ attention within the last decade as Keddie and Mills (2019) stated procedures pertaining to accountability in educational settings have taken various forms ranging from development of standardized tests to the enhancement of measures related to school quality among which principal and teacher performance indicators and professional standards are observable. Performance on such measures is regarded as the main indicator of school success. By the same token, Tuinamuana (2011) believed that “in order for any system of professional teacher standards to raise quality, there must be a strong commitment from the profession to this system” (p. 76). Below, some of the studies done on the notion of accountability in educational settings are covered.

Concerning the importance of educational accountability in academic settings in which teachers are to be accountable towards their students and their profession, Bandele and Ajayi (2013) constructed and validated an accountability scale focusing on such factors as “quality of output, discipline, provision of infrastructure, resource utilization and human development” (p. 695). The outcome was a valid and reliable instrument for measuring educational accountability in rural and urban schools. Although in the same domain as the present research, Bandele and Ajayi (2013) work differs in scope and content.

In the same line, Doğan and Aypay (2016) explained that an accountability behavior is a necessity for improving any organization including higher education institutions. Following the standard-based movement of accountability in attaining relevant promotions, they categorized their 39-item scale into financial accountability, academic accountability, administrative accountability, responsibility, transparency, responsiveness, and explanation and subsequently proved its reliability and validity through employing factor analysis. Again, carried out in the field of accountability, this study has fundamental differences with the present one regarding the components it focused on.

In a more recent study, Karataş et al. (2020) gave prominence to the role school counselors play in academic context and the knowledge, competencies, skills, and attitudes they are obliged to have so that they would be able to organize and evaluate school counseling programs. Highlighting the significance of psychometric properties, the researchers developed a school counselor accountability scale consisting of 54 items. They also divided the scale into four different subcategories of preventive, supportive, developmental, and remedial services and further established the reliability and validity of the instrument. Regarding the ones who need to be accountable in educational contexts, this exploratory research aimed at developing a questionnaire to assess school counselor accountability and did not take teacher accountability into account.

Yet, another study to be worth mentioning is that of Rosenblatt (2017) in which the researcher developed a two-dimensional scale for school administrators and school teachers’ accountability based on external and internal elements indicating a personal disposition to be regarded accountable in work environment. Goal orientation, teacher work performance, and work ethic are examples of the factors which were considered in the scale made. Though the study concentrated on the concept of administrators and school teachers’ accountability, there was no consideration of to whom and how teachers have to be accountable and this forms the essence of the current study.

As observed, there were a good number of studies on the concept of accountability. However, no study was performed to exclusively address teachers’ professional accountability with respect to the responsibilities they have towards students, parents, schools, society, and the profession itself. Accordingly, developing such an instrument may provide better insights regarding the domain of their responsibility so that they may take better decisions to improve their own career.

Being more specific with this inventory of teachers’ accountability and its different aspects, the components are reviewed. The first component of the questionnaire, that is teachers’ accountability to students, requires teachers to support students affectionately and intellectually by building good rapport, using various methods, assessing students objectively, and so forth. Tamir (2020), in this regard, claimed that in the new age of accountability, faculty’s effort to establish good rapports and strong relationships is recognized by the students as the foundation of their growth. Moreover, student voice is viewed as an effective factor in improving the teaching and learning quality as from accountability perspectives, it could enhance students’ gratitude towards their teachers and teaching and learning process. It improves teacher-students’ collaboration and develops students’ perceptions of themselves as learners (Keddie 2015). Olusola Bolarinwa Adeniyi et al. (2019) also found that in cases where students’ perception of teacher accountability is low significantly, teachers do not, indeed, contemplate students’ progress based on their capacity and this impedes achieving optimal learning. Improving students’ attainments, developing their personalities, being available, and helping them understand topics of high perceived difficulty are envisaged to be professionally given more consideration by accountable teachers.

The second component (i.e., teachers’ accountability to parents) primary puts teachers’ responsibility to share succinct report of students’ academic achievement with their parents and keep in touch with them. Accountability to parents, according to Rosenblatt and Wubbels (2021), hinges on the extent to which parents are recognized by teachers to possess the natural right to be given reports on their children’s educational achievements. Deploying the external accountability scale that takes teachers’ accountability to parents as well as elements like feedback, performance reports, and performance evaluation, which were among factors considered in this study as well, into account, they indicated that teachers with more work experience showed higher levels of accountability disposition to parents.

Next, teachers’ accountability toward school was considered. It is believed that dynamic healthy educational system generate parties accountable to one another in a way that it would result in the success of both parties (Holloway 2021). Just as school leadership is accountable for learning outcomes of both teachers and students, so are teachers accountable to school leaders. In an attempt to modify the existing accountability models, Chandran (2021) did a case study in India to observe the impact of accountability systems on teachers’ identities and to illustrate the differences between the models of good teaching imposed by the institution and the ones held by teachers themselves. The results implied that the accountability models needed to be reformed as teachers had a better way of teaching based on which the cultural-political conception of pedagogy is incorporated. Similarly, others (Ekundayo et al. 2018; Omodan and Tsotetsi 2019) regarded teachers as a pivot of any sort of educational system transmitting intellectual skills and molding individuals’ personalities.

Teacher accountability to the society as the fourth components of the questionnaire involves ethical aspects and social affairs of the profession. Respecting teachers’ accountability to the society, Mullai (2017), in a qualitative study, interviewed with five teachers with different years of employment in a focused group. Naming teachers as “moral agents,” the researcher claimed that teachers are the key agents who bring change in societies through using information and communication technologies. Kanika (2016) believed that teaching, with its essential role in human development activities, is a noble profession and Ekundayo et al. (2018) expressed that teachers are among the main individuals who determine the future of humankind as they influence individuals both at schools and in society.

Another critical aspect teachers’ accountability pertains to their sense of responsibility to the profession development as Olusola Bolarinwa Adeniyi et al. (2019) stated teachers are obliged to constantly take part in seminars and workshops to renovate their teaching activities. However, this should not result in as increase in accountability pressures of teacher education programs. Tamir (2020), in this case, conducted a long-term in-depth self-evaluation inquiry through semi-structured interview with individuals taking part in two teacher preparation programs to scrutinize the graduates’ perceptions of the effective characteristics of teacher education programs, the quality of the program faculty, and the teachers’ sense of preparedness for teaching. The outcomes provided some guidelines for teachers’ to deal with such pressures. Teacher education programs, thus, should entail not only professional coursework but a clinical component related to mentored internship (Ronfeldt and Reininger 2012).

## Concluding remarks

High-quality educational settings and students’ success are so inextricably interwoven that one can hardly conceptualize students’ success without taking the role of educational setting into account. In this regard, many organizations and people should cooperate to achieve the desired outcome. For instance, governments are expected to be accountable for appropriate funding of schools that lack sufficient resources. Schools are obliged to be accountable for presenting high-quality education both by providing enriched programs and courses and by employing certified teachers. Teachers are also reckoned to be accountable for having high expectations for every student (Greenblatt et al. 2020). Accordingly, developing an evaluation inventory for assessing teachers’ professional accountability lies at the heart of this research. The study led to the development of a questionnaire with a final version consisting of 29 items across five constructs namely accountability to students, parents, school, society, and profession which were scored on four-point Liker-scale ranging from 1 to 4 indicating the degree the statement was reckoned to be true for the respondent teacher.

The developmental journey of teacher education is not a linear or one-sided direction but an ongoing multidimensional process requiring both pre-service and in-service teachers to improve from all perspectives. It is hoped that the questionnaire developed be beneficial for researchers and practitioners in providing tools for gathering data and exploring patterns underlying individual teachers’ professional identity. Furthermore, with the advent of the post-method era, teacher education models have shifted toward inquiry-oriented ones focusing on developing and training self-determining and self-directing teachers who monitor, reflect, and reform their classroom procedure if necessary. The self-report scale may be used as a yardstick to help teachers assess their perceived professional accountability. Additionally, as “teachers bridge the gap between planned curriculum as espoused in curriculum policy documents and actual curriculum as practiced in actual teaching and learning” (Maphosa et al. 2012, p. 548), school principals and administrators may use the instrument to choose more responsible teachers who strive to promote not only their own knowledge and profession but also the whole education system. Individuals involved in teacher education programs can make use of such a scale to find out more about the amount if teacher familiarity and devotion to their responsibilities and therefore work more on the weak parts.

Even though the established methods were followed in designing and validating this self-report scale, the limitations addressed below are noteworthy to be taken into account as they might have implicitly or explicitly influenced the results. First, although the researchers utilized inductive and deductive methods for domain selection and item generation to comprehensibly verify the conceptualization of teachers’ professional accountability, some intervening issues were out of their control for practical and time limitations. For example, conducting interviews with more proficient people (i.e., teacher educators), having a larger sample size, and conducting the study nationwide could lead to better results. Hence, other researchers may replicate and gain a deeper understanding of teachers’ professional accountability as undoubtedly there would be other perspectives worth to enquire. Besides, the qualitative section of this study was predominantly conducted through a comprehensive review of the literature and the implication of the structured interviews. Missing from the current study is the usage of data triangulation and various sources of data collection as moving beyond interviews may open new venues for the research and unveil some other domains. By observing teachers’ and students’ engagement, creating focus groups and casual chatting with teachers, documenting, journaling, and so many other sources, future studies are recommended to reflect on and reveal different aspects of teachers’ professional accountability. Moreover, it must be emphasized that this research take no account of individual differences into consideration as the conduction of the study was coincided with the spread of coronavirus in public and certain research procedures such as questionnaire piloting were done online. In this circumstance, it was not feasible for the researchers to gather more data from a larger sample size or to pay attention to the participants’ individual differences. Thus, further studies that control the influence of individual differences may result in shedding light on how these factors may contribute to teachers’ professional accountability.

## Data Availability

Data is available if asked by emails from the corresponding author.

## Appendix

**Table 5:** Teachers’ professional accountability

A	Accountability towards students				
1	I assist students in attaining educational goals by providing maximum learning opportunities.	1	2	3	4
2	I provide students with the information they require and the materials they need.	1	2	3	4
3	I have an excellent rapport with students and behave all students patiently and equally well.	1	2	3	4
4	I use various methods, techniques, and resources to improve students' performance.	1	2	3	4
5	I encourage a sense of hard work in students and motivate them to strengthen their week points.	1	2	3	4
6	I assess students' performance objectively.	1	2	3	4
**B**	**Accountability towards Parents**				
7	I have a sense of respect for parents.	1	2	3	4
8	I keep in touch with parents and inform them about students' academic performance.	1	2	3	4
9	I carefully listen to parents and help them with students' affairs.	1	2	3	4
10	I make a relationship with parents based on trust and understanding.	1	2	3	4
11	I work well with parents to enhance students' progress.	1	2	3	4
12	I give parents a report on the way they can accomplish their duties.	1	2	3	4
**C**	**Accountability towards School**s				
13	I cooperate well with the administrative system.	1	2	3	4
14	I am punctual and well-disciplined	1	2	3	4
15	I enable schools to provide high-quality education and achieve their objectives.	1	2	3	4
16	I adhere to the principles and rules of schools	1	2	3	4
17	I demonstrate decent personal behavior and attitude towards school principals and staff.	1	2	3	4
18	I provide school principals with a report on students' performance and inform them bout students' wants and needs.	1	2	3	4
**D**	**Accountability towards Society**				
19	I teach students to act according to moral standards and cultural issues of society.	1	2	3	4
20	I motivate low socioeconomic sections of society to educate.	1	2	3	4
21	I endeavor to eliminate violence, discrimination, and social ills and bring equality	1	2	3	4
22	I coordinate and participate in different social affairs	1	2	3	4
23	I dedicate myself to students' social and emotional development	1	2	3	4
24	I strive to provide deprived students with updated technology, libraries, laboratories, and other school resources.	1	2	3	4
**E**	**Accountability towards the Profession**				
25	I try to have lifelong learning to guarantee professional development	1	2	3	4
26	I work collaboratively with colleagues and help them improve.	1	2	3	4
27	I attempted to have sufficient knowledge and skills and take courses if necessary.	1	2	3	4
28	I promote the teaching profession by doing research.	1	2	3	4
29	I attend seminars, workshops, and other enrichment programs to be up-to-date.	1	2	3	4
30	I dedicate myself to keep the quality of the profession high.	1	2	3	4

This instrument is composed of 30 statements concerning teachers’ professional accountability. Please indicate the degree to which each statement applies to you by marking whether you:

**Never = 1; Sometimes = 2; Often = 3; Always =4**

## Abbreviations

TEFL: Teaching English as a foreign language
Qual: Qualitative
Quan: Quantitative
SPSS: Statistical Package for Social Sciences
KMO: Kaiser-Meyer-Olkin
